# Exploring Community Mental Health Systems – A Participatory Health Needs and Assets Assessment in the Yamuna Valley, North India

**DOI:** 10.34172/ijhpm.2020.222

**Published:** 2020-11-23

**Authors:** Kaaren Mathias, Meenal Rawat, Anna Thompson, Rakhal Gaitonde, Sumeet Jain

**Affiliations:** ^1^Herbertpur Christian Hospital, Dehradun, India.; ^2^Sir Edmund Hillary Marg, New Delhi, India.; ^3^Sree Chitra Tirunal Institute for Medical Sciences and Technology, Thiruvananthapuram, India.; ^4^Department of Social and Political Science, University of Edinburgh, Edinburgh, UK.

**Keywords:** Participatory, Needs Assessment, India, Mental Health, Community, Health Systems

## Abstract

**Background: **In India and global mental health, a key component of the care gap for people with mental health problems is poor system engagement with the contexts and priorities of community members. This study aimed to explore the nature of community mental health systems by conducting a participatory community assessment of the assets and needs for mental health in Uttarkashi, a remote district in North India.

**Methods: **The data collection and analysis process were emergent, iterative, dialogic and participatory. Transcripts of 28 in-depth interviews (IDIs) with key informants such as traditional healers, people with lived experience and doctors at the government health centres (CHCs), as well as 10 participatory rural appraisal (PRA) meetings with 120 people in community and public health systems, were thematically analysed. The 753 codes were grouped into 93 categories and ultimately nine themes and three meta-themes (place, people, practices), paying attention to equity.

**Results: **Yamuna valley was described as both ‘blessed’ and limited by geography, with bountiful natural resources enhancing mental health, yet remoteness limiting access to care. The people described strong norms of social support, yet hierarchical with entrenched exclusions related to caste and gender, and social conformity that limited social accountability of services. Care practices were porous, pluralist and fragmented, with operational primary care services that acknowledged traditional care providers, and trusted resources for mental health such as traditional healers (malis) and government health workers (accredited social health activists. ASHAs). Yet care was often absent or limited by being experienced as disrespectful or of low quality.

**Conclusion:** Findings support the value of participatory methods, and policy actions that address power relations as well as social determinants within community and public health systems. To improve mental health in this remote setting and other South Asian rural locations, community and public health systems must dialogue with the local context, assets and priorities and be socially accountable.

## Background

**Key Messages**

** Implications for policy makers**

- Global mental health programmes and policy can increase relevance and acceptability by engaging and assessing community level needs and assets for mental health.
- Use of participatory methods for health needs assessment can increase dialogue, engagement and participation of civil society and community health system actors with the formal health system.
- To increase access to care for people with mental health problems national policy can work synergistically with non-biomedical practitioners, such as traditional healers or pharmacists.
- Routinely collecting and disaggregating data on social determinants among mental health service users such as caste, religion, gender, age and education status can better quantify demographics and social risk factors of service users, and ultimately increase equitable outcomes.

** Implications for the public**

This study documents a participatory assessment of mental health in the Yamuna valley, in remote North India conducted by a non-profit organisation. The aim was to identify needs and assets in formal and community health systems. We found strong community assets such as mutual social support, shared belief systems, and trusted village-based traditional healers (malis) and government health workers (accredited social health activists, ASHAs) who provided care and mediated help-seeking. There were also excluding hierarchies related to gender and caste. In the formal health system, strengths included operational primary care services and practitioners using pluralist culturally acceptable approaches, but there were no services for primary or secondary mental healthcare, and examples of absent or low-quality care. Increased mental health in this setting requires increased social inclusion linked to gender and caste, increased community participation in social accountability, increased dialogue and training between community and health providers, and between traditional, allopathic and informal providers.

In India mental illness accounts for the largest portion of the disease burden, yet more than 80% of those affected do not access care.^1,2^ With 1.3 billion people, 630 districts and 22 official languages, there are diverse contexts and systems, and a key component of this ‘mental healthcare gap’ is poor system responsiveness to the contexts and needs of community members.^1,3,4^ Identifying and describing community mental health needs and assets can increase partnerships, responsiveness and community agency to allow excluded citizens to shape systems and policies.^5-7^ Describing the resources communities draw on to respond to mental ill-health acknowledges communities as experts in their own right, and as active agents promoting ‘community mental health competence.’^4^ Ongoing contact with and use of trusted local providers (both allopathic, traditional and complementary systems) can increase accessibility and acceptability of healthcare facilities.^7,8^ Other benefits include bi-directional flows of knowledge, contextually relevant interventions, partnerships between local and global actors,^4^ programmes responsive to community priorities^9,10^ greater family involvement, and economic benefits.^8^

In this study, set in India, the ‘community mental health system’ is considered as the collective of the social and political determinants of health and relationships at the community level along with Indian state health systems players including ASHAs (accredited social health activists).^6,11,12^ The key public mental ill-health response, the National Mental Health programme (NMHP) focuses primarily on access to psychiatric care and medicines, facets which have seen significant progress and roll-out in the past 5 years.^13,14^ Government, academia and civil society have called for strengthening the NMHP by integrating mental health with community health systems and platforms such as non-communicable diseases, acknowledging the importance of social determinants of mental health.^1,15-20^ Seeing community health systems as wider than government cadres allows a more relevant assessment of local contexts, and identifying and collaborating with community resources is essential for effective delivery of psychosocial therapies.^21^

Community engagement and social accountability are central to universal health coverage in India,^22-24^ and the NMHP has always had community participation as a key (but as yet not operationalised^19^) component. However, almost no community mental health programmes anywhere have used participatory health needs/asset assessments to inform design of contextually relevant programs and policies. There is little guidance and few or no concrete examples on how to do this.

 This study uses participatory methods to answer the following research question: what are the assets, needs and priorities for community mental health in the upper Yamuna valley, North India?

## Methods

###  Theoretical Framework

For this study we adapted and merged the theoretical approaches of community health needs assessment^25^ and participatory asset mapping^7,9,26^ using participatory rural appraisal (PRA) as a bridge to facilitate this.^27^ Asset based approaches, which document resources and focus on strengths to enhance and preserve rather than deficits to be remedied^9^ are often seen as an alternative to needs-based (‘deficit’) approaches, and thus with different starting points. In bringing the 2 approaches together, we sought to avoid a binary that sees all communities as self-sufficient or all health services as deficient, and instead, to construct community health systems in a holistic way with both strengths and needs. PRA is an approach that empowers local research participants to generate, analyse and own research data and uses methods such as participatory mapping, transect walks, well-being grouping and seasonal calendars.

###  Setting

This study is set in the upper Yamuna valley which includes the administrative block of Naugaon, district Uttarkashi, in the north Indian state of Uttarakhand. An overview of the study area (Table 1), shows a highly rural and disadvantaged demographic profile.

**Table 1:** Sociodemographic Profile of the 3 Study Districts and National Comparison Data^28^

**Indicator**	**India National**	**Uttarakhand State**	**Uttarkashi District**	**Naugaon Block**
Total population	1200 million	10.1 million	330 000	60 000
% Population rural	72.2	69.5	92.6	100.0
% Population under 18 years	34.9	28.9	29.7	-
% Population Dalit (SC)	16.6	18.8	24.4	29.1
Sex ratio (female to 1000 males)	940	963	1031	969
Literacy (% literate female)	65.5	70.1	62.3	40.0
Literacy (% literate male)	82.1	87.4	88.8	59.0
Maternal mortality	178	292	158	-

Abbreviation: SC, scheduled caste.

This mixed methods study is set in the remote and mountainous Yamuna valley in Uttarakhand state where people have practiced subsistence agriculture for generations, with income derived from religious pilgrimages more recently. Census classifications are General (most advantaged), Other Backward castes (more dominant) and Scheduled castes (SCs)/Tribes (Dalits and indigenous, typically most disadvantaged). Caste operates most strongly in spheres of food, marriage and in religious practices and traditional Hinduism dominates.^29^ The dominant patriarchy favours men and disadvantages women.^29^ The study was led by Burans, a project team based at the non-profit Herbertpur Christian Hospital collaborating with district functionaries of the Department of Health and Family welfare. The identity and stance of researchers is as follows: KM is a New Zealand public health physician who has lived in India for 23 years and speaks fluent Hindi. MR is of Scheduled Tribe identity from Uttarakhand. AT is a New Zealander who has lived many years in South Asia and RG and SJ are researchers of Indian origin.

The NMHP has been in operation since 2016^30^ but the nearest government psychiatrist is 6 hours travel away. Burans team visited government facilities on 12 occasions to find very limited or no essential psychotropic medicines during this study period. In response to Burans team advocacy, a primary care government doctor started a monthly mental health clinic at the government health centre (CHC) from October 2019. A total of 57 patients attended the clinic in the first 4 months.

###  Participatory Data Collection and Analysis

This participatory health needs and assets assessment broadly followed the first 3 of the 4 key steps typical of this methodology^10^: (*a*) define participatory health needs and assets assessment parameters and objectives; (*b*) describe community needs and assets, and health services; (*c*) analysis; and (*d*) coproduce programme design and adaptation.

(*a*). In the first step, the Burans advisory group (which includes community members who are ‘experts by experience’) proposed the broad questions of this study in early 2019 and the research protocol and ethics application were built from these questions. Researchers (KM and MR) and members of the Burans Yamuna valley team, (19 of 22 are permanent residents in Yamuna valley) acted as the steering group, clarifying the objectives of the research and participating in a 3-day training in PRA methods.

(*b*). In the second step, the team collected both quantitative (service delivery) and qualitative data between July 2019 and March 2020, prior to the incidence of any coronavirus disease 2019 (COVID-19) cases in Uttarakhand state. The quantitative data was collected by project staff of Burans and followed a simple survey tool to enumerate public, private, informal and traditional health services and providers and service utilisation. The majority of in-depth interviews (IDIs) and focus group discussions (FGDs) were audio-recorded, supplemented by note taking and photos of visual outputs. Interviews followed a semi-structured interview guide that probed broad understandings of mental health and illness and their causes, help-seeking, practices for promoting mental health and assets and needs for mental health. Further data was collected through the PRA technique in groups ranging in size from 5 to 10 and documented and notes taken in a research journal by MR and KM, as well as project reports.

(*c*). In the third step, analysis of both quantitative and qualitative data was conducted in dialogue with community representatives and involved translating and transcribing interviews, FGDs and PRA workshops. MR discussed the original Garwhali words with community members, and data were analysed inductively and thematically using a validated approach inspired by Braun and Clarke.31 Additional data sources such as pictures and visual diagrams generated during PRA exercises plus team reports and research journals (participant observation by KM and MM) were also used to generate categories and themes. AT, MR and KM coded the same 5 interviews and compared and contrasted coding to develop a coding framework for all data sources to yield 753 codes and 93 categories in discussion with MR and KM. Quantitative data was reviewed and discussed with Government functionaries and workers, and descriptive tables generated to summarise key findings. For discrepancies encountered between government online and field data, we prioritised data verified in the field.

(*d*). MR and AT conducted member checking meetings with groups of ASHA workers, community members and CHC staff as well as people with lived experience in November 2019 by presenting findings pictorially or diagrammatically so community members could engage with and prioritise the relevance of findings. Revised categories were grouped into 10 themes by MR, AT and KM and themes were further refined in dialogue with community and health systems participants, Burans advisory group members and with the involvement of supportive national government players, in order to leverage their ‘authorising credibility.’

###  Participants

A total of 148 residents in the Yamuna valley participated in IDIs, FGDs or participatory appraisal workshops. Key informants (for example traditional healers) were selected purposively while the participants in the ten PRA workshops were selected to represent inter-sectional perspectives such as older men from Dalit (SC) communities and mothers of school-aged children from a Brahmin (General) village or a mixed caste group of young men who play sports together. The source of verbatim quotes is noted with this convention: (sex, age, type of data collected, community role eg, C = community member) except for the relatively few government functionaries, in order to protect their anonymity. The participants and formats of data collection are outlined in Table 2.

**Table 2:** Participants in Data Collection

**Number **	**Identity/Role of Participant**	**Format of Data Collection**	**Setting of Data Collection**
4	Senior government doctors in the district	IDI	Workplace
2	Junior government doctors in the area	IDI	Workplace
1	Private doctor – gynecologist	IDI	Workplace
2	ANM – a village-level health worker	IDI	Community health centers
2	Pandits – involved in astrology, religious activities and rituals	IDI	Respective homes
2	Oracles (*mali ) *	IDI	Burans’ Office/Barkot market
3	Private and public pharmacists	IDI	Workplace
10	Community members with psycho-social disability	IDI	In their homes
2	ASHA supervisor	IDI	At primary healthcare
3	ASHA and AWWs (all female) n = 18 total	FGD	In ASHA members home or AWW centre
10	Groups of community members (9-12 each) n = 102 total	PRA	Public community settings

Abbreviations: ASHA, accredited social health activist; AWW, Anganwadi worker; IDI, in-depth interview; FGD, focus group discussion; PRA, participatory rural appraisal; ANM, auxiliary nurse midwife.

## Results

Ten themes related to assets and needs within community and public health systems in the Yamuna valley were identified and grouped under 3 meta-themes related to place, people and practices. The participatory dialogue during and after data collection moved beyond consultation to dialogue and preliminary partnerships between community participants and the health system. Table 3 summarises the meta-themes, needs and assets in this study.

**Table 3:** Summary of Meta-themes, Needs and Assets Found in This Study

**Meta-theme**	**Needs**	**Assets**
Place	More accessible and coordinated care	Natural resources and beauty provide welcome
People	Social structures that are less hierarchical and excluding	Social support to people with mental health and other problems
	Greater collective action and advocacy	Cultural practices support mental health
Practices	Respect, regulation and quality in services	Pragmatic pluralist practice
		Community and primary health services are operational and accessed

###  Place - Blessed and Limiting

 Participants identified the Yamuna valley as a remote place ‘blessed’ by deities, beauty and natural resources while simultaneously constrained by the sparse primary care and inaccessible secondary health services also determined by the geography. Place-based assets also included religious tourist-linked income and high levels of land ownership.

**“** *May whosoever come, they will be served” – natural resources and beauty provide welcome*

Participants described the valley as ‘devbhoomi’ (land of the gods) with beauty and agricultural productivity as natural assets while the source of the sacred Yamuna river provides economic benefits from pilgrimages. Both men and women described stopping to appreciate natural beauty as a strategy to reduce *‘tension’* (stress). Women described social norms of collective work as an opportunity to seek psycho-social support, while appreciating the natural scenery:

*“If we are feeling down we often decide to go with 8 or 10 women to the jungle and we take food with us and half the day we sit, look out at the scenery and take rest and talk [ ]. But they (mother and father in law ) just think we’re collecting firewood the whole day” *(F, 34y, PRA, C).

 Participants also described how the physical location supports a good agricultural income and conveyed a sense of generosity and hospitality in this rural setting that is not found in cities:

*“In cities a poor person has many hardships. Even if you want a glass of water you pay. [ ]. Here it is so pure: the water, weather, everything... May whosoever come, they will be served”* (F, 40y, IDI, C).

####  “We have to travel 6 hours just to get an X-ray” – need for more accessible and coordinated healthcare

 Universally there was priority need for both biomedical and traditional care that was more accessible (both geographically and financially). The remote location limited access to formal health services and participants described high costs of the road journey for diagnostics and secondary care in Dehradun.

 Access to care was complicated further by the highly privatised health system and absence of coordinated care. Help-seeking practices involved traditional healers as the first port of call, because they were closest geographically and payment could be made later or in kind, even if some participants thought biomedicine more effective than traditional healing.

 Many community members did not have a formulated conception of mental healthcare but those who had sought help for family members described essentially non-availability of biomedical services or medicines; and those with symptoms of depression and anxiety did not have an awareness of possible modalities such as psychosocial support or talking therapy.

 The challenges of geography in a large rural district are summarised by a government functionary below:

*“The NMHP is officially sanctioned across Uttarakhand but it is very challenging to get any psychiatrist to live in these remote places. Even if there is, [ ] with poor roads, how can he drive 5 hours to provide care regularly in the Yamuna side? We also have appointed some other officers in the NMHP but as there are no patients coming, they are working more in the non-communicable disease control team*” (IDI, government functionary).

 People described changing social patterns and a breakdown in traditional norms, such as drug addiction and urban migration, as problems of place (eg, tourist linked income providing cash for drugs) and both seen as fast-moving, disruptive and needing resources and responses at the level of state policy.

###  People – Both Tightly Knit and Hierarchical

 The community is linguistically and culturally homogeneous, evidenced by uniform attire according to age and gender, universal participation in community festivals, and widely shared beliefs and social norms that, in part, limit participation in collective social action. When community members were probed about why they had not protested about poor quality or absent health services they stated they had not thought of this, and that it had not been done before. Description of rules limiting eating food, entry into temples and women asking permission to spend money or move freely, underlined the steeped hierarchies privileging men and the General (advantaged) caste.

Assets identified included high and growing levels of education among young people, growing contact with *bahr (*‘the outside’) via internet and social media, and a moderately active *panchayati raj* (administrative 5 membered village councils).

####  “People help each other” – support that is widespread but eroding to people with mental and other health problems 

 The benefit of a tight knit community is high levels of social support within a village or extended family, although Dalit participants described this as occurring primarily within traditional caste groupings. A young man belonging to a dominant caste described how support is need-based and includes domestic and agricultural realms:

*“Here [in the Yamuna valley] people help each other. Like when all the people in a household are sick or have some problem, other people in that community will take care of their children or cut their grass ( ) or make their haystacks for them”* (M, 25y, IDI, C).

 However, this support was not universally experienced: women with psycho-social disability described feeling that they were a burden and socially excluded, while others considered social support less prevalent with increasingly individualised and globalised ways of living. A woman recounts loss of once reliable practices of reciprocity and increasingly commodified labour:

*“Even in the last few years I have seen quite a change, like now I think if someone is sick the neighbours will just say ‘You better hire someone to come and work your harvest and pick things up for you. We are too busy ourselves to help here and who knows when we would get the favour returned’”* (F, 35y, FGD, C).

####  “You don’t want to be seen as someone who doesn’t believe in their own culture” – Cultural practices support mental health

A universal shared belief system considered that mental health depended on the ‘will of God.’ The primacy of traditional explanatory frameworks was both socially required and proximate as *devtas* (deities) and *mali*(oracles) are found in nearly every village. This sense of obligation for a man with psycho-social disability is described below:

*“I did it [went to the mali ] like anyone else in our society. [ ] When a pandit (priest) says to do this or do that, you do it. You don’t want to be seen as someone who doesn’t believe in their own culture. People will blame you if you don’t do or follow these traditions” *(M, 45y, IDI, C).

In community mapping exercises to understand help-seeking, ‘in-laws’ (family heads) together with the *mali* or Hindu priests in every group were depicted as most proximate and with the largest circles (using *chappati*(Venn) diagrams), denoting importance. An additional resource for mental health and stress-relief is the almost universal participation in *melas* (religious festivals) held annually in each village, providing an opportunity for social contact, religious rites and community recreation.

####  “What isn’t unfair?” –social structures are hierarchical and excluding, yet also can be supportive

 Social hierarchies limited access to resources and worsened mental health. Women reported lower access to healthcare and that gender relations reduced women’s mental health and wellbeing. The limited freedom of movement and the absence of discretionary income and time for women were frequent themes as exemplified below:

####  Woman 1: “So, what makes me angry is, like, I want to go to my mother’s and my husband won’t permit me to go and it feels so unfair.”

*Woman 2: “What isn’t unfair? The jobs in the house, women do nearly everything like cooking, cleaning, caring for children, caring for animals, caring for mother and father in law. There are a few changes from when I was a child to be honest, although nowadays some men cook a little” *(F, 25y, PRA, C).

 Caste relations were also a key locus of exclusion, although people from General caste were dismissive, describing these relations as long-standing but non-discriminatory. Implicit in their dialogue was minimising of their own privilege, while acknowledging caste-related ‘pollution of food’ and associated discrimination as illustrated below:

*“(We) people do not eat any food prepared by Dalit, if they invite us to any function there will be cooks from outside or we will prepare our own food. Everybody supports each other in our village regardless of caste, but these are old traditions which are followed since ages”* (M, 45y, IDI, C).

 Dalit people (SC) described how exclusion impacted all spheres of their lives, for example, impacting friendships and their choices of education:

*“Actually, most of us prefer to send our children to the town or even to Dehradun. They (our children) are treated better there. Actually, it is more that we want them to believe in themselves. Here in the village schools somehow, they get this idea that they cannot do things and that nothing can change. Even in Naugaon or Barkot (larger towns in the Yamuna valley) at school they feel better about themselves and so we mostly try to send our kids there if we can” (*M, 27y, PRA, C).

 Yet while gendered social structures were unfair, participants described how they also supported mental wellbeing. Men described going to town with other male friends, playing carom (table-top game), cards or sports games to reduce stress, while young men described mental health benefits from time alone doing physical exercise.

 Women also reduced their stress by spending time with other women while working together. One woman showed agency by using her limited discretionary time to seek psychosocial support below:

*“Yes, [when we go to cut grass] actually this is often the time when we talk as we go and while we are walking there or back. We talk while we work or sometimes, we work side by side and help each other to finish our work. Talking to each other also helps in passing time and our minds feel lighter ( mann halka ho jata hai )”* (F, 36y, PRA, C).

####  We cannot speak out as it may cause us problems – need for greater collective participation and advocacy

 Participants agreed there was generally little collective social or political activism in the valley. People with Dalit identity risked their ongoing relationships if they publicly voiced their wishes, such as to enter the local temple. Participants described a sense of community loyalty and the possibility of offending someone’s relative as important disincentives to speaking out:

*“Actually, the people in that village will not speak out or say anything. They cannot as it is a member of their own village and they know them very well and then there could be problems. But everyone knows who is lazy or active, of course”* (Government functionary).

When discussing this finding during member checking with government health service providers, participants critically reflected in a lively dialogue that nothing can change ‘if we stay quiet’* (‘agar hum choop raithe hai ’*). After this discussion one functionary proposed writing a letter of complaint about the minimal initiative from Government to fill key vacancies in the local health services.

###  Practices – Porous, Pluralist and Fragmented

 People in Yamuna Valley access multiple sources of help for mental health problems including community/family social support, biomedical care, and traditional healers. This study uncovered a range of traditional and allopathic systems with porous access and boundaries and pragmatic referrals between different traditional practitioners; and public health systems that were operational but limited in their responsiveness (to emerging and disruptive problems), coordination and quality of care.

####  “People go back and forth, and we work with that” – a pragmatic, pluralist practice

 The ability of community members to actively navigate different care systems (even within the limits of the social hierarchies described) suggested that people intrinsically understand the value provided by different providers so that ‘shopping around’ becomes an appropriate response to bridging ‘treatment gaps’ and limitations in both biomedical and traditional healing systems. Care providers, on the other hand, indicated openness to different healing traditions, encouraging people to move across and between providers, as a government doctor outlines below:

*“Well of course devi and devta are the most important in this area. We cannot do anything without the patient needing to consult with them and also deciding whether to follow allopathic advice or not. [ ]. If we speak against it even fewer people will seek care so we try to acknowledge that Bhagwan (the Lord) is overall but that medicines can also be helpful [ ]. I would say around 50% of people come first to the CHC and maybe 50% go first to consult with the gods or mali. [ ] So, people go back and forth and we just have to work with that” *(Government functionary).

While junior doctors described initial discomfort with the ‘dawa aur dua’ (prayers and medicines) approach, they were also and encouraged by seniors to positively acknowledge local belief systems and simultaneously recommend bio-medical treatment. *Malis* and other traditional religious healers were equally pragmatic in their approach and referred people who were not improving for allopathic treatment. Community members did not demarcate allopathic and traditional systems and also did not differentiate between public and private providers. ASHA workers were viewed as a key community source of advice on how to navigate the public health system. Visual *chapatti* (Venn) diagrams (Figure) depicted ASHA workers as the provider closest to the family, but used small circles to denote their role, suggesting they did not influence larger domains. This representation of the help-seeking and referral practices shows that Malis, with ASHAs, are the most proximate community health system carers, and as dominant mediators of help-seeking, also illustrating the pragmatic referral systems between public and private, formal and informal, traditional and biomedical systems.

**Figure:**
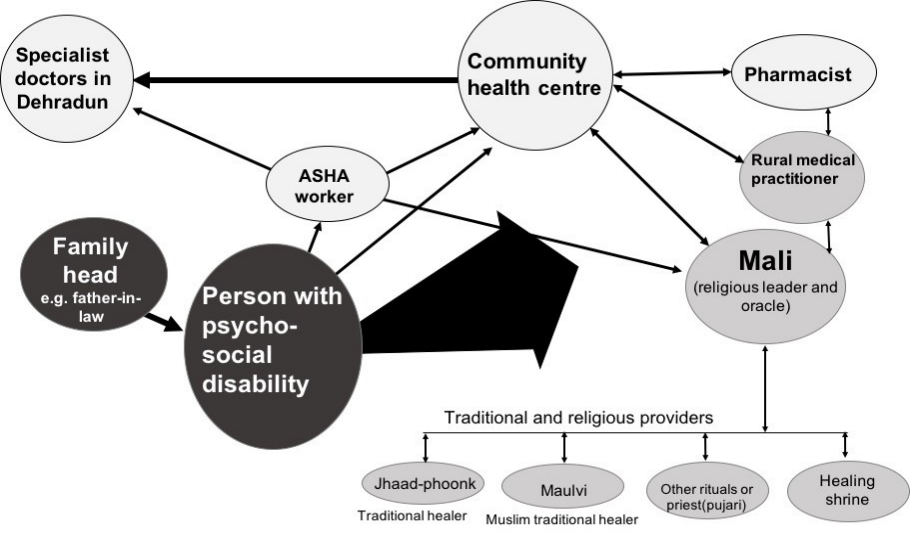
Help-Seeking and Referral Practices in the Yamuna Valley for People With Psychosocial Disability. The large black arrow denotes help-seeking by the affected person with all providers collectively. Abbreviation: ASHA, accredited social health activist.

####  “The doors are open daily” – the community providers and primary government health services are operational and accessed

Two government community health centres serve the Yamuna valley, with each having an average of 40-45 out-patients daily, respectively. The Uttarkashi District Hospital is 4 hours’ drive away and roughly 50% of sanctioned doctor positions across the district are filled. Supplementary file 1 provides further details on health services in Uttarkashi district.

Community members described basic primary health services as functioning and staffed daily. The CHC staff referred patients with problems like stress to the government homeopathic doctor at the CHC but also described the need for formal counselling support and services. When first asked about mental health needs, participants out forward the desire for locally available computerised tomography scans and specialist hospitals. However, after dialogue and reflection in PRA workshops, an ASHA worker proposed greater knowledge as a higher need, and after discussion, community members ranked increased knowledge and awareness (*jagrukta*) as the greatest mental health need. Knowledge and training were also identified as a high priority by ASHA workers.

####  “We don’t even sit when we see the doctor” – The need for respect, regulation and quality in services

 Participants highlighted frequent experiences of poor quality health services, unaddressed staff absenteeism and inconsistent provision of services. Health functionaries who participated in the study ascribed the poor quality of services to limited accountability structures:

*“There is no point saying anything, as things will continue the way they are [ ]. Others also know so why should I say anything? But of course, ‘ vyavastha honi chaiye ’ (there should be proper systems in place). [ ]. Even though we have equipment like X-Ray machines, there is no surgeon. Many positions are vacant. Actually ‘ aaspital khud bimarhai ’ *(the hospital itself is unwell)” (IDI, government functionary).

 Participants described health services as doctor-focussed, with a hospital visit taking at least half a day (travel and waiting) with little confidentiality. For example, outpatient consultations took place with up to ten other patients also standing around the patient. History taking was completed in one or 2 minutes and, on occasion, the doctor would write and hand over a prescription without telling a patient the diagnosis or giving instructions about what to do next. No participants perceived a difference in treatment related to caste or gender.

 Patients, in turn, expressed low regard and respect for the doctors, describing them as motivated by personal financial gain when they routed essential Government issued medicines through commercial pharmacies or choosing to refer most maladies:

*“These CHC doctors cannot do anything much. They just write on the slip that we should go to Doon hospital and send us off. Even a small fracture they don’t attend to, and unless the senior doctor is there, nothing to be gained by going to CHC” *(M, 50y, FGD, C).

## Discussion

 This study documented multiple ‘assets’ for mental health in the Yamuna valley particularly for those already advantaged in the social hierarchy (eg, General caste and male). However, modern bio-medical resources for mental health are almost non-existent for all, and especially for more marginalised people, even if there are significant community resources available and used by people from all social identities. Nuances are evident in each of the 3 meta-themes, with assets and needs emerging sometimes in a tension, such as the remote location which supports both the closely knit communities as well as prevalent hierarchies that exclude. The study also revealed the complex adaptive processes individuals and communities adopt, and some of (albeit limited) ways that systems of healing (traditional and bio-medical) have adapted to these realities.

Participatory methods like PRA helped this study ask more relevant questions through the action, reflection cycle, and supported shared learning among research participants ^32^. For example, through dialogue, community members prioritised the need for mental health awareness above specialist diagnostic services. Participatory approaches surfaced the importance of context, for example, in the pragmatic engagement with prevailing explanatory frameworks by traditional healers and allopathic doctors.^18,33^ The participatory dialogue led to the establishment of preliminary partnerships, as observed elsewhere.^5-7^ The findings surfaced and also underline the complexity of power dynamics in the health system, such as for ASHA workers who are central in help-seeking but with little power.^11^ Data collection by community-based team members increased dialogue and included voices of those typically excluded.^32^

This study approached participation from the framing provided by the decolonization debate, which recognises that dominant research and healthcare paradigms, supported as they are by powerful global institutions and interests, tend to ‘force fit’ communities into a constrained paradigm that preserves the status quo.^34^ By considering participation as a liberatory praxis,^35^ we propose that actively involving communities in research can serve to question dominant perspectives, while potentially improving access to mental health services and enabling better mobilisation of the existing resources available to a particular community. A recent publication set in India, provides an excellent framework to ensure research with communities is both participatory and uses ‘horizontal’ approaches to reduce power differentials between communities and researchers.^36^

The study identified the centrality of medical pluralism in help-seeking and the nature of care across biomedical and traditional providers. The high levels of agency demonstrated by people with mental distress and their families in moving between and negotiating their way through different systems is described elsewhere,^37^ as are the positive attitudes of biomedical and traditional carers towards each other, documented in other parts of India and beyond.^38-40^ These attitudes reflect the idea of co-existence (dawa and dua). However, underpinning this are unequal power relations, shaped by how the state has constructed traditional healing practices as irrational and illegitimate, seeking to regulate these practices legally and through attempts at formal integration.^39-41^

The notion of the ‘treatment gap’ as used in global mental health often solely focusses on availability and access to professionalized biomedical and psychosocial care^42^ but neglects formal and informal sources of care such as traditional healers, non-registered medical practitioners, pharmacists, community and family support.^40,41^ We found people’s help-seeking practices lead to an informal integration (through ‘shopping around’) of the available social, biomedical and psychological resources. These diverse and informally integrated care systems may provide ‘access’ to different yet complementary forms of care that support well-being. For example, some traditional healing spaces may enable people with psychosocial disabilities to live well with a long-term mental health problem, and with family support, may facilitate positive social outcomes such as increased social participation and inclusion. However, there are limitations to an informal integration premised on pluralistic notions of ‘dawa aur dua.’ Further data is needed to understand on what basis people make decisions, their expectations from care systems, and the challenges faced in navigating these systems. A crucial question is how communities can be best supported to use existing care and support resources appropriately. The answers may lie in participatory methods to enable dialogues about the ‘value’ of different care systems, such as the shift in community felt needs from computerised tomography scanners to knowledge, that occurred in this study.

The hierarchies operating in the culturally homogeneous society of Yamuna valley that particularly disadvantage women and Dalit people were evident in this study. The critical consciousness and occasional critique of these hierarchies displayed by community members is itself an asset. Intersectionality acknowledges that social and structural determinants of mental ill-health compound for individuals. Other studies have found economic status, gender, education, social status (registered caste or tribe), and age are important inter-related structural determinants in understanding equity in India.^43,44^ Global mental health has paid insufficient attention to inequity related to intersectional identities.^4,45^ Future research is needed to develop processes for in-depth analysis of how intersectional layering manifests in individual lives, and ways to challenge intersectional gender and other power relations in order to develop more strategic, effective and equitable programmes and health systems.^46^

Systems are nuanced, complex and display contradictions. Fracturing social forces such as individualisation^47^ and globalisation (associated with education, internet connectivity, cash incomes in cities) have mixed benefits and feedback loops. Western derived mental health therapies that focus on the individual may be the problem and not solution in India.^47^ One facet, such as strong social cohesiveness can simultaneously limit another facet such as willingness to engage in advocacy or protest using mechanisms of social accountability. While community engagement is regarded as good practice there is a risk that this can be used as a pretext to shift responsibility for complex mental health problems to households and communities without ensuring they are supported with required resources.

This study adds to the voices of many others identifying problems in the public health system in rural India which include under-resourcing, lack of accountability and regulation, gender inequity, corruption, poor quality and disrespectful care, the proliferation of vertical programmes that compromise universal healthcare and insufficient remuneration of ASHAs.^6,11,22,23,48^ Despite the seeming intractability of these challenges, this study describes participatory processes and pathways through which public health and community systems can dialogue to support and strengthen mental health. A possible limitation was that the research was conducted through a non-profit organisation which could lead to social desirability bias. There was limited data collected from complementary providers, as this was not permitted by the Uttarakhand state Department of Ayurveda and other traditional therapies (Department of AYUSH).

###  Implications

Implications of this study are relevant for India’s NMHP in remote districts such as Uttarkashi. Firstly, the NMHP needs to be adapted for the local context, or even better, co-produced with community members to build on local assets. Currently the model of mental healthcare in India’s NMHP is uniform, despite the highly diverse settings and needs of communities.^13,17,19,49^ In the Yamuna valley, the actual practice of doctors showed some responsiveness to the prevailing belief systems through a pragmatic and pluralist practice. Nationally, the NMHP could be adapted to increase cultural competencies and relevance in both community and public health systems through dialogue with, and training of traditional, allopathic and informal providers (such as pharmacists).^41,50,51^ The NMHP and mental health services nationally could build on the widely available asset of natural environments across rural India, to ease mental distress by including interventions such as green exercise and therapy building in natural spaces,^52^ while taking into account structural obstacles such as limited freedom of movement for women in North India for this.^53^

Secondly, this study underlines the importance of social determinants such as gender and caste, for mental health. There have been wide calls for the health policy nationally,^54^ and specific programmes such as the NMHP to increase focus on social determinants and equity in mental health,^19,49^ Possible policy responses include facilitating engagement between civil society, researchers and policy-makers to understand and theorise action on social determinants, equity and policy action in mental health in the State^54-56^; and ensuring monitoring of the NMHP includes measures of equity such as access to care and use of essential medicines among people such as migrant labourers, people with disabilities and Dalit people.^55,57^

Thirdly, this study illustrated the need to better build community mental health competencies as part of the NMHP^17^ such as increasing knowledge, social accountability and safe social spaces (social inclusion)^4^ for example by increased safe social spaces for women and Dalit, and modelling non-hierarchical relationships within the health sector^38^ and building into the NMHP governance and performance measures, ways to include social accountability and community engagement.^49^

## Conclusion

 Participatory assessment for mental health in the Yamuna valley surfaced important needs and assets in both community and public health systems. In this setting there is informal integration of the available social, biomedical and psychological resources that for many supports positive mental health even with limited access to biomedical care. Participatory dialogue surfaced as a strategy to ensure communities are empowered to engage with existing care and support resources appropriately. Findings support policy actions that address power relations as well as the intersectionality of social determinants within community and public health systems. To improve mental health in this remote setting and similar settings across the diverse land of India, community and public health systems must be socially accountable and responsive to the local context and priorities.

## Acknowledgements

 Appreciation to Jeet Bahadur, Jeph Mathias, Atul Goodwin Singh, Manoj Singh, Kiran Chauhan, Minika Devi, Prakash Lal, all the Burans employed community workers as well as Sana Parakh and Tanushree Joshi who supported data collection. Thanks also to support from Dr. Joshi, Chief Medical officer for Uttarkashi, Dr. Nidhi and Dr. Rana and all members of the CHC teams at Naugaon and Barkot.

## Ethical issues

 The study was approved by the Emmanuel Hospital Association’s Institutional Ethics Committee in August 2019 and assigned protocol number 208.

## Competing interests

 The authors declare that none of them have any conflict of interest in the publication of this paper. KM and MR report receiving grants from Mariwala Health Initiative, during the conduct of the study which contributed towards their salaries and time given to the conduct of this study.

## Authors’ contributions

 KM conceived of the paper, participated in data collection, analysis and wrote the first draft, MR conducted data collection and contributed to analysis, AT led data analysis, RG supported literature review and wrote sections of discussion, SJ contributed to the literature review and sections of discussion. All authors contributed to repeated drafts of the paper.

## Funding

 This research was carried out with funding from the Mariwala Health Initiative, Mumbai, India.

## Authors’ affiliations

^1^Herbertpur Christian Hospital, Dehradun, India. ^2^Sir Edmund Hillary Marg, New Delhi, India. ^3^Sree Chitra Tirunal Institute for Medical Sciences and Technology, Thiruvananthapuram, India. ^4^Department of Social and Political Science, University of Edinburgh, Edinburgh, UK.

## Supplementary files


Supplementary file 1 contains Table S1.
