# Supplementary material for: Exploring Community Mental Health Systems – A Participatory Health Needs and Assets Assessment in the Yamuna Valley, North India
Source: Int J Health Policy Manag. 2020 Nov 23;11(1):90–9. doi: 10.34172/ijhpm.2020.222 (PMC9278393; doi:10.34172/ijhpm.2020.222)
Supplement: Supplementary file 1 — contains Table S1. [file ijhpm-11-90-s001.pdf]

Supplementary file 1

Table S1. A Summary of Public Health Services in Uttarkashi, Uttarakhand

Health Service Resource	Uttarkashi District	Naugaon Block	Observations
Number of district hospitals	2	0	
Number of CHC	4	2	
Number of PHC	10	1	
Number of sub-centres	82	19	
Number of psychiatrists	0	0	Total n= 7 government psychiatrists in Uttarakhand state
Number of doctors trained for NMHP	2	0	
[redacted]			
CHC – Naugaon	Mean outpatients per day	40	
	NMHP medicines stocked on assessment visit Feb 2020	0	
	Total no. of doctors in Naugaon	06	Number of unfilled positions, n = 14 doctors
CHC – Barkot	Mean outpatients per day	45	
	NMHP medicines stocked on assessment visit Feb 2020	0	
	Total number of doctors in Barkot	5	Number of unfilled positions, n=3 specialists
ANM	Total number of ANM in Naugaon block	09	Number of ANM in Uttarakhand, n= 1825 out of 2283 sanctioned
ASHA	Total number of ASHAs in Naugaon block	128	Number of ASHA in Uttarakhand, n= 11573 out of 11651 sanctioned
